# Improved phyllosphere microbiome composition of tea plant with the application of small peptides in combination with rhamnolipid

**DOI:** 10.1186/s12866-023-03043-0

**Published:** 2023-10-23

**Authors:** Hao Chen, Yujie Song, Shuangshuang Wang, Kai Fan, Hui Wang, Yilin Mao, Jie Zhang, Yang Xu, Xinyue Yin, Yu Wang, Zhaotang Ding

**Affiliations:** 1 Tea Research Institute, Qingdao Agricultural University, Qingdao, 266109 China; 2 Tea Research Institute, Shandong Academy of Agricultural Sciences, Jinan, 250100 China; 3 Rizhao Tea Research Institute, Rizhao, 276827 China

**Keywords:** *Camellia sinensis* (L.) O. Kuntze, Phyllosphere, Small peptides, Surfactants, Rhamnolipid, Microorganisms

## Abstract

**Background:**

Small peptides play a crucial role in plant growth and adaptation to the environment. Exogenous small peptides are often applied together with surfactants as foliar fertilizers, but the impact of small peptides and surfactants on the tea phyllosphere microbiome remains unknown.

**Results:**

In this study, we investigated the effects of small peptides and different surfactants on the tea phyllosphere microbiome using 16S and ITS sequencing. Our results showed that the use of small peptides reduced the bacterial diversity of the tea phyllosphere microbiome and increased the fungal diversity, while the use of surfactants influenced the diversity of bacteria and fungi. Furthermore, the addition of rhamnolipid to small peptides significantly improved the tea phyllosphere microbiome community structure, making beneficial microorganisms such as *Pseudomonas*, *Chryseobacterium*, *Meyerozyma*, and *Vishniacozyma* dominant populations.

**Conclusion:**

Our study suggests that the combined use of small peptides and surfactants can significantly modify the tea phyllosphere microbiome community structure, particularly for beneficial microorganisms closely related to tea plant health. Thus, this preliminary study offers initial insights that could guide the application of small peptides and surfactants in agricultural production, particularly with respect to their potential for modulating the phyllosphere microbiome community in tea plant management.

**Supplementary Information:**

The online version contains supplementary material available at 10.1186/s12866-023-03043-0.

## Introduction

Small peptides (Small molecule oligopeptides) have been extensively used in various fertilizers due to their crucial roles in maintaining plant growth and development, aiding plant adaptation to the environment, promoting plant metabolism, and inducing plant immune responses [1–4]. The effects of small peptides on microorganisms are multifaceted. Small peptides can improve the resistance of plants, make plants easier to form symbiotic relationships with some beneficial microorganisms, promote their growth and reproduction in plants, and also regulate and activate the immune system of plants, and inhibit the growth and reproduction of harmful microorganisms [5–9]. Importantly, small peptides also serve as nutrients to provide nourishment for microorganisms. Our previous studies have demonstrated that small peptides can enhance the ability of tea plants to resist abiotic stress by increasing the activity of antioxidant enzymes and regulating hormone and amino acid metabolism pathways [10]. However, the effect of small peptides on tea phyllosphere microorganisms has not been reported.

Rhamnolipid and sophorolipid are non-toxic, harmless, edible, and degradable biosurfactants that are commonly employed in conjunction with foliar fertilizers to enhance their efficacy [11–14]. Rhamnolipid is a biological metabolic substance produced by *Pseudomonas* or *Burkholderia*, which exhibits inhibitory effects on the growth and reproduction of certain plant pathogenic bacteria and fungi [15]. Moreover, it can serve as a natural carbon source, providing energy for the metabolism of dominant microorganisms, thereby augmenting their numbers and activity [16, 17]. Sophorolipid, on the other hand, represents a class of microbial secondary metabolites produced by *Candida* through a specific fermentation process utilizing sugar and vegetable oil as carbon sources [18–20]. Although its impact on microorganisms is similar to that of rhamnolipid, its selectivity towards bacterial and fungal communities differs [12]. Tween 20, a chemical surfactant widely employed in agriculture, also exerts a significant effect on microorganisms [21, 22]. However, the effect of the combination of small peptides and surfactants on the phyllosphere microbiome remains unknown, particularly in terms of their effects on beneficial microorganisms and plant pathogens.

Tea phyllosphere microorganisms are an important part of tea plant growth, which are closely related to the health of tea plants. Similar to soil microorganisms, the study of phyllosphere microorganisms is equally important. Stimulating the maximum potential of phyllosphere microorganisms and utilizing them can bring agricultural production to a new stage. Studies have shown that the development-specific metabolites in tea plants drive the assembly of functional phyllosphere microbial communities and their biological functions of continuously inhibiting fungal diseases [23]. At the same time, studies have also revealed the basic mechanism of microbiome assembly in tea plants and the potential impact of microbiome-mediated resilience frameworks on leaf homeostasis [24]. However, for tea plants, there are still few studies on phyllosphere microorganisms.

In this study, small peptides were used alone or in combination with three surfactants (chemical surfactants and biosurfactants) to explore the effects of small peptides and surfactants on the phyllosphere microbial community of tea plants. We speculate that the use of small peptides and surfactants will significantly change the community composition of tea phyllosphere microorganisms, especially beneficial microorganisms or pathogens closely related to tea tree health. Through this study, we give the optimal combination of small peptides and surfactants in the application of tea plants and can provide a reference for the regulation of the phyllosphere microbial community in tea tree planting management.

## Results and analysis

### OTUs cluster analysis of phyllosphere bacteria and fungi

**Fig. 1:**
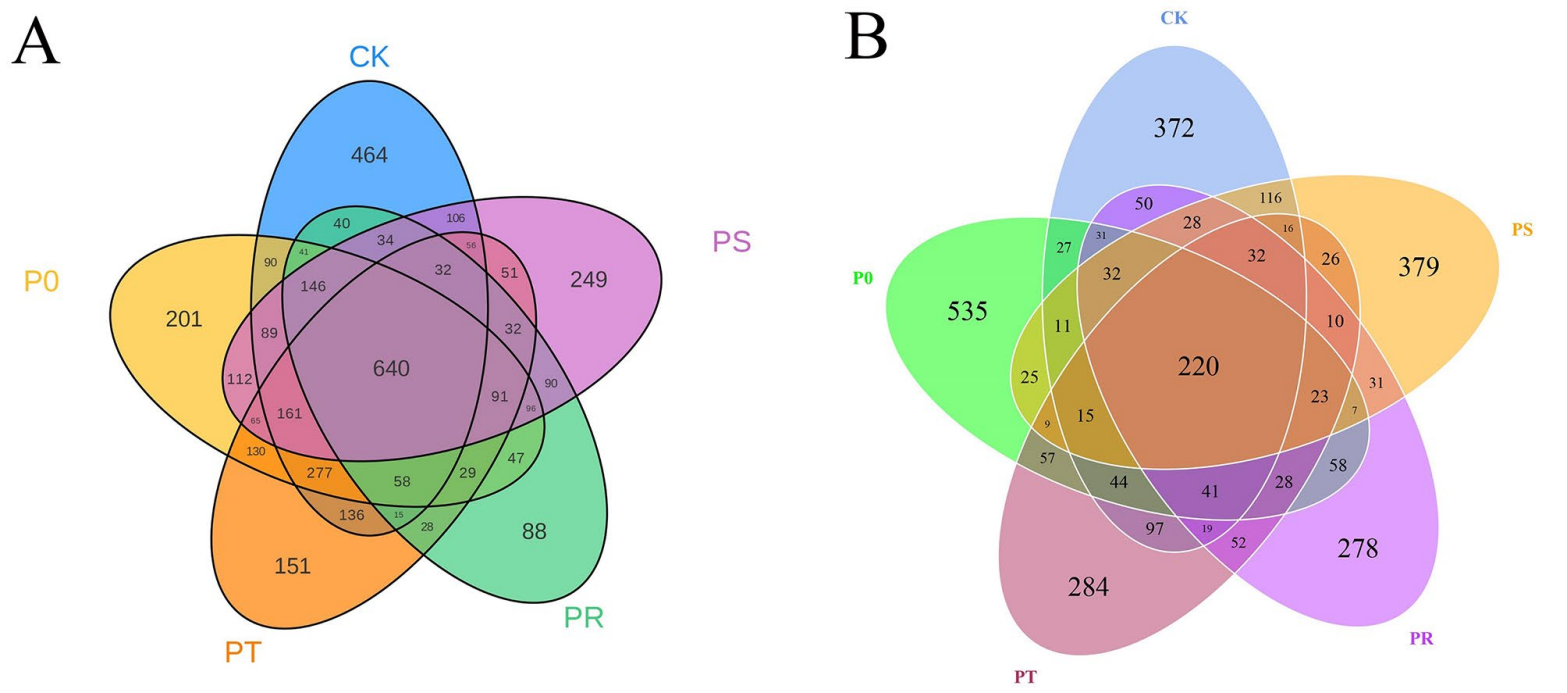
Venn diagram of bacteria in each group based on OTUs(A). Venn diagram of fungi in each group based on OTUs(B). Foliar spraying with pure water (CK). Foliar spraying with small peptides (P0). Foliar spraying with small peptides and Tween-20 (PT). Foliar spraying with small peptides and rhamnolipid (PR). Foliar spraying with small peptides and sophorolipid (PS).

To analyze the species diversity of bacteria and fungi in tea phyllosphere after the application of small peptides and surfactants, and to better demonstrate the species differences between different treatments, the effective data obtained were subjected to OTUs (Operational Taxonomic Units) clustering analysis, and Venn diagrams were generated. As shown in Fig. 1A, there were 640 common OTUs among the tea phyllosphere bacteria in different treatments, of which 464 were unique to the CK, 201 to the P0, 151 to the PT, 88 to the PR, and 249 to the PS. As shown in Fig. 1B, there were 220 common OTUs among the tea phyllosphere fungi in different treatments, of which 372 were unique to the CK, 535 to the P0, 151 to the PT, 88 to the PR, and 249 to the PS.

### Diversity analysis of bacteria and fungi in phyllosphere

To analyze the richness, diversity, and community composition of leaf-associated microbial communities under different treatments, we conducted alpha diversity and beta diversity analyses (Figure S1). The Shannon index reflects the total number and proportional abundance of classifications in a sample, with higher community diversity indicating a more even species distribution and larger Shannon index. The Chao1 index estimates the total number of species in the community sample. The Shannon index and Chao1 index of P0 bacteria were significantly higher than those of CK, while the Shannon index and Chao1 index of P0 fungi were significantly lower than CK. The use of small peptides resulted in a decrease in bacterial diversity and an increase in fungal diversity. Additionally, we performed UPGMA cluster analysis based on the OTU-weighted UniFrac distance, where greater distance indicates greater differences in community structure among the treatments.

### Cluster analysis of phyllosphere bacteria and fungi at the genus level

**Fig. 2:**
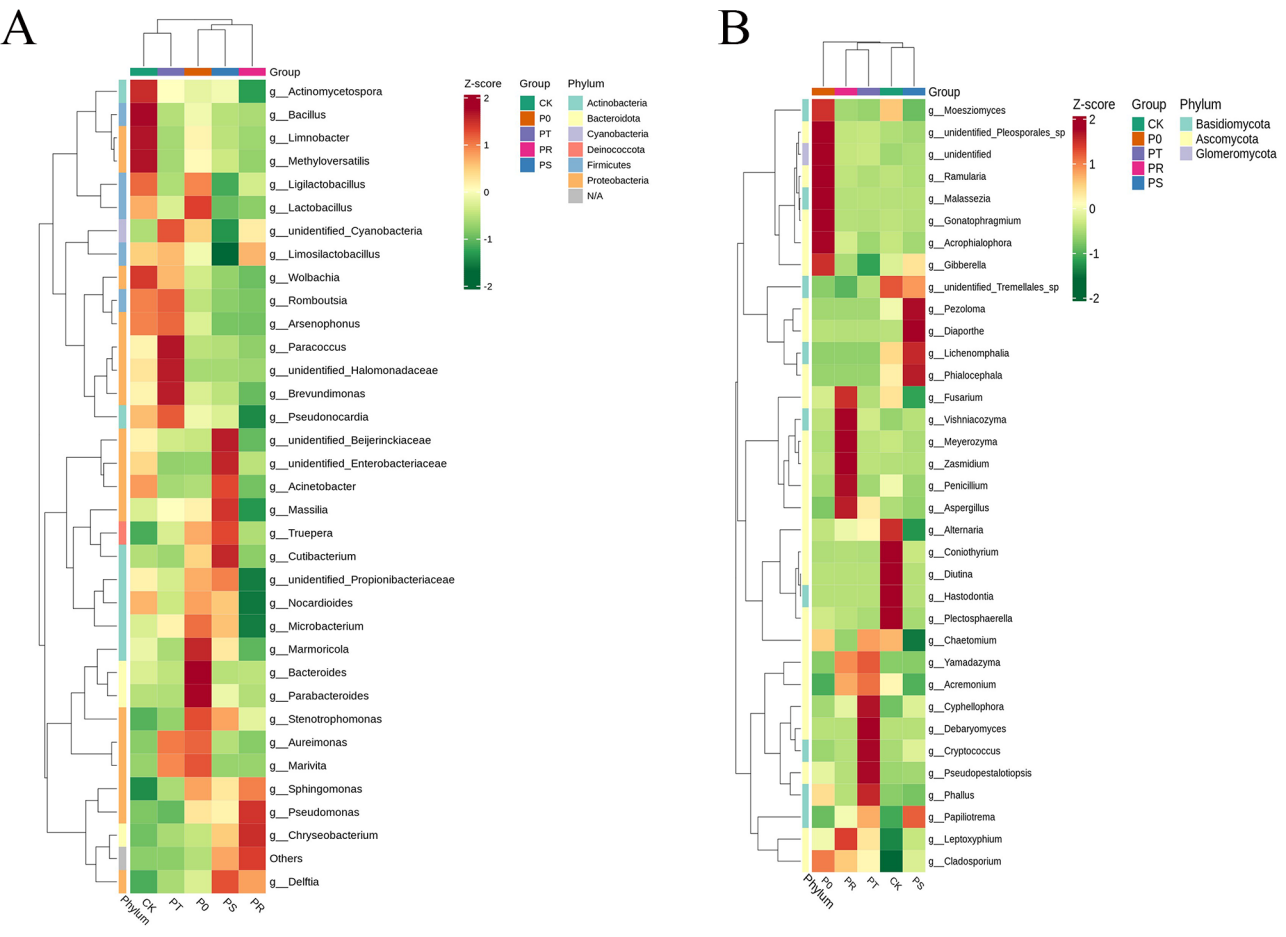
Cluster heat map of species abundance of bacteria in each group at genus level(**A**). Cluster heat map of species abundance of fungi at genus level(**B**). The vertical is the grouping information, the horizontal is the species classification information, and the clustering tree in the figure is the genus-level clustering tree; the values corresponding to the heat map are Z-Score standardized relative quantitative data

To analyze the differences in the composition of the phyllosphere microbiome after the application of small peptides and surfactants, we conducted clustering analysis of bacteria and fungi at the genus level. As shown in Fig. 2A, the bacterial community of CK was mainly composed of *Bacillus*, *Limnobacter*, *Methyloversatilis* and *Actinomycetospora*, while that of P0 was dominated by *Bacteroides*, *Parabacteroides*, *Marmoricola* and *Lactobacillus*. The bacterial community of PT was mainly composed of *Paracoccus*, *unidentified Halomonadaceae* and *Brevundimonas*, while that of PR was dominated by *Pseudomonas* and *Chryseobacterium*. The bacterial community of PS was mainly composed of *unidentified Beijerinckiaceae*, *unidentified Enterobacteriaceae*, *Acinetobacter*, *Massilia*, *Truepera* and *Cutibacterium*. As shown in Fig. 2B, the fungal community of CK treatment was mainly composed of *Plectosphaerella*, *Coniothyrium*, *Diutina*, and *Hastodontia*, while that of P0 was dominated by *Acrophialophora*, *Ramularia*, *Malassezia* and *Gonatophragmium*. The fungal community of PT was mainly composed of *Cyphellophora*, *Debaryomyces*, *Cryptococcus*, *Pseudopestalotiopsis* and *Phallus*, while that of PR was dominated by *Meyerozyma*, *Zasmidium*, *Vishniacozyma*, *Penicillium* and *Aspergillus*. The fungal community of PS was mainly composed of *Pezoloma*, *Diaporthe*, *Phialocephala* and *Lichenomphalia*. Our results indicate that the dominant populations of microorganisms in each treatment were different, suggesting that small peptides and different surfactants have different selectivity towards bacteria and fungi.

### Difference analysis of phyllosphere microbial community structure between each treatment group and control group

#### Analysis of bacterial community structure differences

**Fig. 3:**
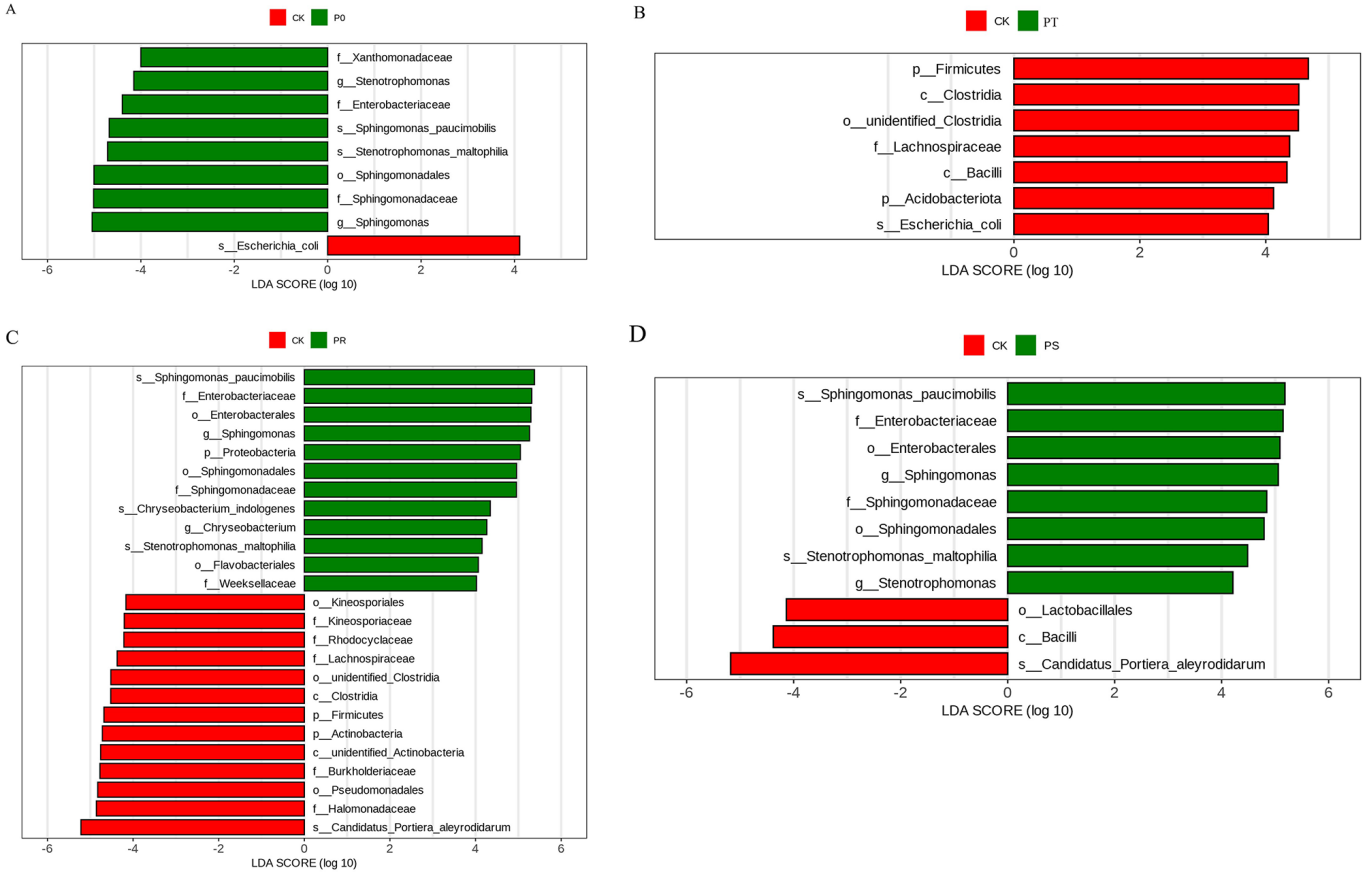
Populations with significant difference in bacterial abundance between CK and P0(**A**). Populations with significant differences in bacterial abundance between CK and PT(**B**). Populations with significant differences in bacterial abundance between CK and PR(**C**). Populations with significant difference in bacterial abundance between CK and PS(**D**). The LDA value distribution histogram shows the species whose LDA Score is greater than the set value (default setting is 4), that is, biomarker with statistical differences between groups. The length of the bar chart represents the degree of influence of different species. The longer the length, the greater the contribution of the species to the difference between groups

To better compare the biologically relevant differences in phyllosphere bacterial community composition between the treatment groups and the control group after the application of small peptides and surfactants, we used LEfSe (LDA Effect Size) to identify statistically significant biomarkers. There were 9 biomarkers that significantly differentiated the bacterial community structure between CK and P0, of which *Xanthomonadaceae*, *Stenotrophomonas*, *Enterobacteriaceae*, *Sphingomonas paucimobilis*, *Stenotrophomonas maltophilia*, *Sphingomonadales*, *Sphingomonadaceae*, and *Sphingomonas* were dominant in P0, while *Escherichia* was dominant in CK (Fig. 3A). There were 7 biomarkers that significantly differentiated the bacterial community structure between CK and PT, of which *Firmicutes*, *Clostridia*, *unidentified Clostridia*, *Lachnospiraceae*, *Bacilli*, *Acidobacteriota*, and *Escherichia* were dominant in CK, while there were no dominant populations in PT (Fig. 3B). There were 25 biomarkers that significantly differentiated the bacterial community structure between CK and PR, of which *Sphingomonas paucimobilis*, *Enterobacteriaceae*, *Enterobacterales*, *Sphingomonas*, *Proteobacteria*, *Sphingomonadales*, *Sphingomonadaceae*, *Chryseobacterium indologenes*, *Chryseobacterium, Stenotrophomonas maltophilia*, *Flavobacteriales*, and *Weeksellaceae* were dominant in PR, while *Candidatus Portiera aleyrodidarum*, *Halomonadaceae*, *Pseudomonadales*, *Burkholderiaceae*, *unidentified Actinobacteria*, *Actinobacteria*, *Firmicutes*, *Clostridia*, *unidentified Clostridia*, *Lachnospiraceae*, *Rhodocyclaceae*, *Kineosporiaceae*, and *Kineosporiales* were dominant in CK (Fig. 3C). There were 10 biomarkers that significantly differentiated the bacterial community structure between CK and PS, of which *Sphingomonas paucimobilis*, *Enterobacteriaceae, Enterobacterales, Sphingomonas, Sphingomonadaceae, Sphingomonadales, Sphingomonas paucimobilis*, and *Stenotrophomonas* were dominant in PS, while *Candidatus Portiera aleyrodidarum, Bacilli*, and *Lactobacillales* were dominant in CK (Fig. 3D). Our results suggest that the combination of small peptides and rhamnolipid has the most significant impact on the bacterial community in the tea phyllosphere.

#### Analysis of fungal community structure differences

**Fig. 4:**
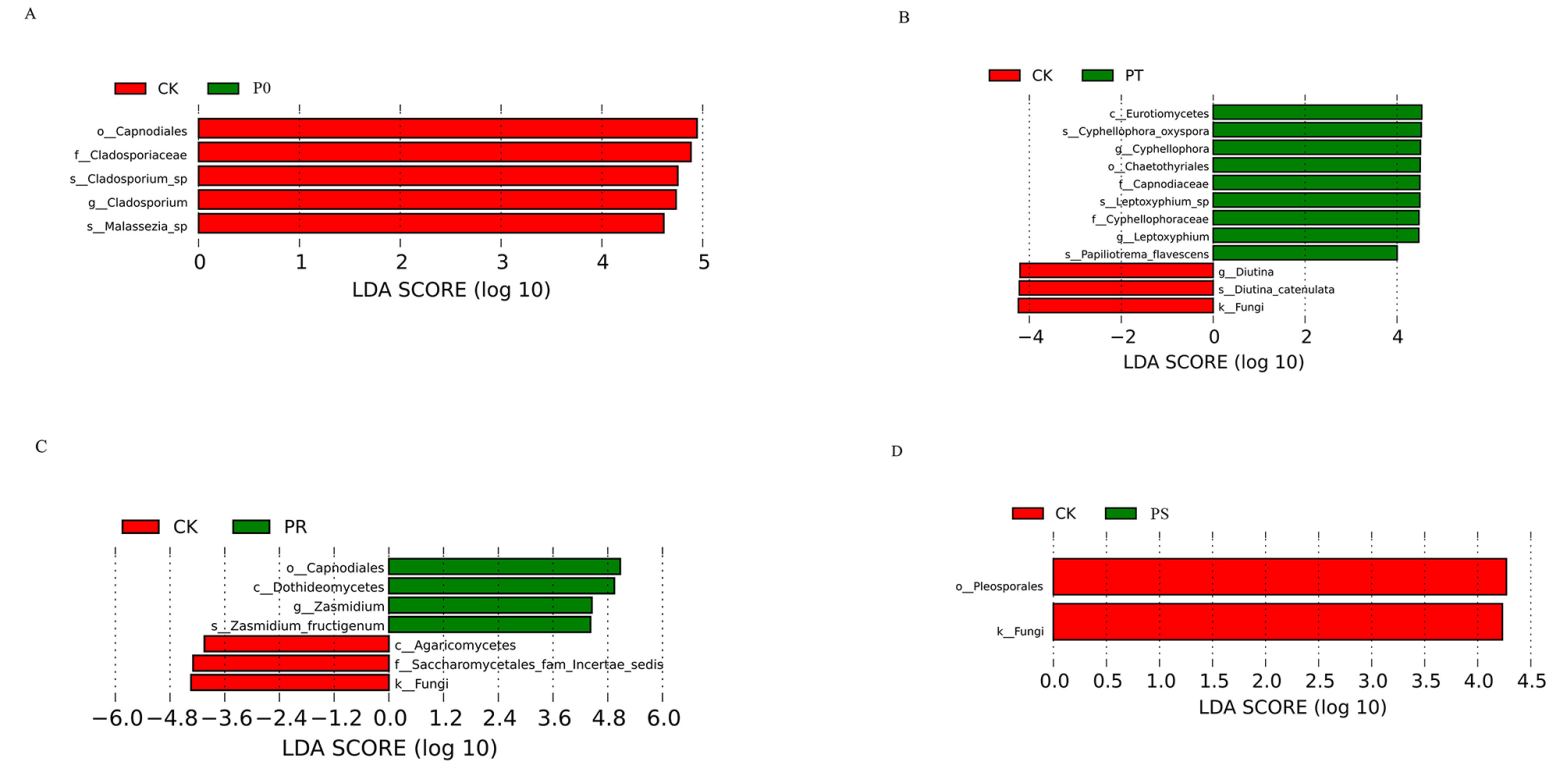
Populations with significant difference in fungal abundance between CK and P0(**A**). Populations with significant differences in fungal abundance between CK and PT(**B**). Populations with significant difference in fungal abundance between CK and PR(**C**). Populations with significant difference in fungal abundance between CK and PS(**D**). The LDA value distribution histogram shows the species whose LDA Score is greater than the set value (default setting is 4), that is, biomarker with statistical differences between groups. The length of the bar chart represents the degree of influence of different species. The longer the length, the greater the contribution of the species to the difference between groups

To better compare the biologically relevant differences in phyllosphere fungal community composition between the treatment groups and the control group after the application of small peptides and surfactants, we used LEfSe (LDA Effect Size) to identify statistically significant biomarkers. There were 5 biomarkers that significantly differentiated the fungal community structure between CK and P0, of which *Capnodiales, Cladosporiaceae, Cladosporium sp, Cladosporium*, and *Malassezia sp* were dominant in P0, while there were no dominant populations in CK (Fig. 4A). There were 12 biomarkers that significantly differentiated the fungal community structure between CK and PT, of which *Eurotiomycetes, Cyphellophora oxyspora, Cyphellophora, Chaetothyriales, Capnodiaceae, Leptoxyphium sp, Cyphellophoraceae, Leptoxyphium*, and *Papiliotrema flavescens* were dominant in PT, while *Diutina* and *Diutina catenulata* were dominant in CK (Fig. 4B). There were 6 biomarkers that significantly differentiated the fungal community structure between CK and PR, of which *Capnodiales, Dothideomycetes, Zasmidium*, and *Zasmidium fructigenum* were dominant in PR, while *Saccharomycetales fam Incertae sedis* and *Agaricomycetes* were dominant in CK (Fig. 4C). There were 2 biomarkers that significantly differentiated the fungal community structure between CK and PS, of which *Pleosporales* was dominant in CK, while there were no dominant populations in PS (Fig. 4D). Our results suggest that PT and PR have the most significant impact on the fungal community in the tea phyllosphere.

### Effects of spraying small peptides and surfactants on the microbial function of tea leaves

#### Relative cluster analysis of phyllosphere bacterial function

**Fig. 5:**
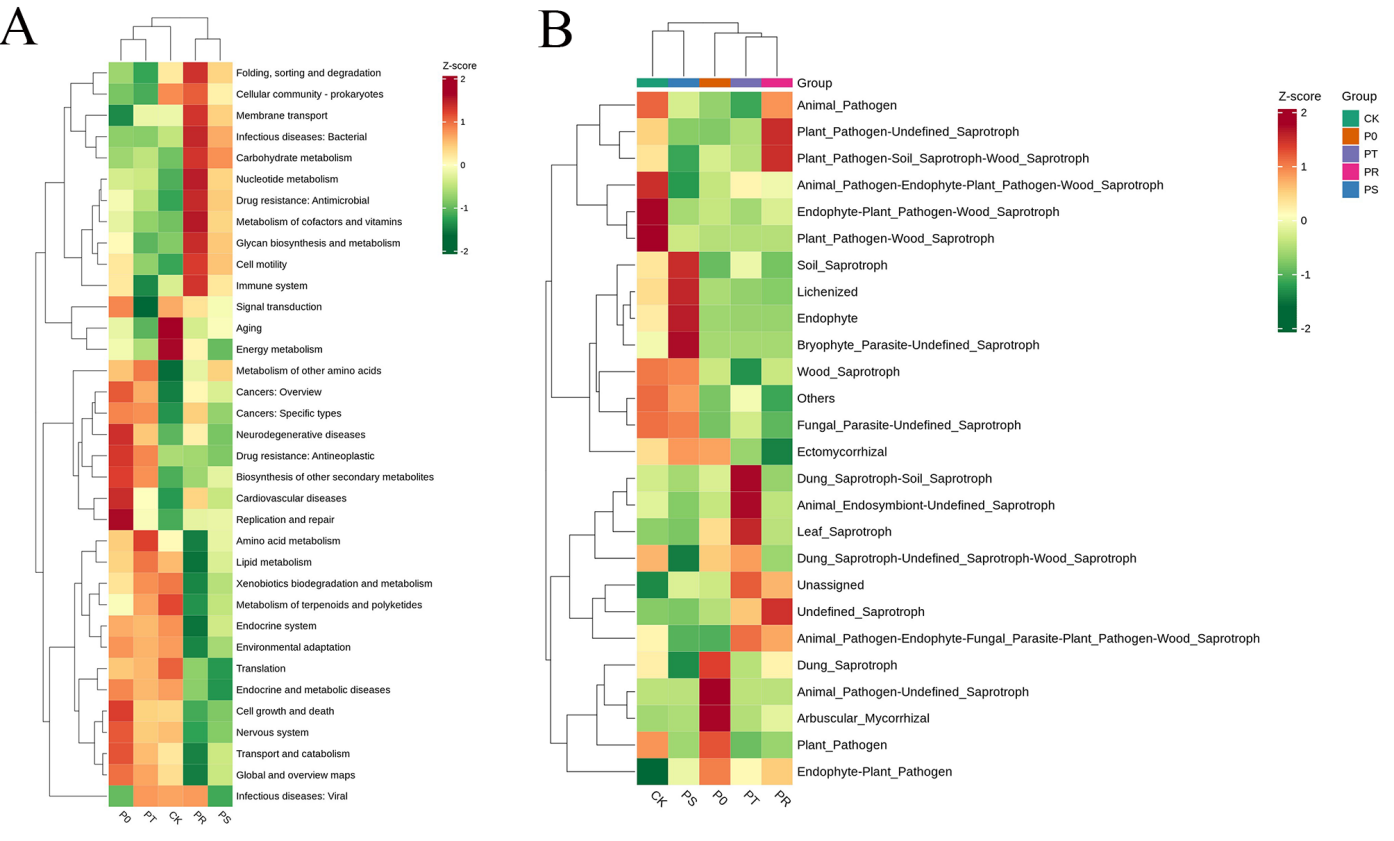
Tax4Fun2 functional annotation clustering heat map of bacteria based on OTUs between groups(A). FunGuild functional annotation clustering heat map of fungi based on OTUs between groups(B). Horizontal represents functional annotation information, vertical represents sample information, and lattice represents relative abundance. The redder the color, the higher the relative abundance, and the bluer the relative abundance

To analyze the impact of the application of small peptides and surfactants on the functional profile of bacterial communities in the tea phyllosphere, we used Tax4Fun2 to annotate and cluster their functional profiles. As shown in Fig. 5A, the bacterial functions in CK were mainly enriched in Aging, Energy metabolism, Metabolism of terpenoids and polyketides, Translation, and Cellular community – prokaryotes. The bacterial functions in P0 were mainly enriched in Replication and repair, Biosynthesis of other secondary metabolites, and Cell growth and death. The bacterial functions in PT were mainly enriched in Amino acid metabolism, Lipid metabolism, and Metabolism of other amino acids. The bacterial functions in PR were mainly enriched in Folding, sorting and degradation, Cellular community - prokaryotes, Membrane transport, Nucleotide metabolism, Metabolism of cofactors and vitamins, Glycan biosynthesis and metabolism, Cell motility, and Immune system. The bacterial functions in PS treatment were mainly enriched in Carbohydrate metabolism.

#### Relative cluster analysis of phyllosphere fungi function

To analyze the impact of the application of small peptides and surfactants on the functional profile of fungal communities in the tea phyllosphere, we used FunGuild to annotate and cluster their functional profiles. As shown in Fig. 5B, the fungal functions in CK were mainly enriched in Endophyte-Plant Pathogen-Wood Saprotroph, Plant Pathogen-Wood Saprotroph, and Animal Pathogen-Endophyte-Plant Pathogen-Wood Saprotroph. The fungal functions in P0 were mainly enriched in Animal Pathogen-Undefined-Saprotroph, Arbuscular Mycorrhizal, and Ectomycorrhizal. The fungal functions in PT treatment were mainly enriched in Dung Saprotroph-Soil Saprotroph and Phyllosphere Saprotroph. The fungal functions in PR were mainly enriched in Plant Pathogen-Soil Saprotroph-Wood Saprotroph, Plant Pathogen-Undefined Saprotroph, and Undefined Saprotroph. The fungal functions in PS were mainly enriched in Bryophyte Parasite-Undefined Saprotroph, Endophyte, Lichenized, and Soil Saprotroph. These results suggest that the application of small peptides and surfactants significantly impacts the functional profile of fungal communities in the tea phyllosphere, with significant differences observed among the different treatment groups.

## Discussion

After foliar application of small peptides, the number of unique OTUs specific to bacterial communities in the tea phyllosphere was significantly reduced to 201, compared to 464 in the control group. In contrast, the number of unique OTUs specific to fungal communities significantly increased to 535, compared to 372 in the control group. The use of small peptides resulted in the dominance of *Bacteroides, Parabacteroides*, and *Lactobacillus* as bacterial populations, and the dominance of *Acrophialophora, Ramularia, Malassezia*, and *Gonatophragmium* as fungal populations. The fungus *Acrophialophora* has demonstrated potential in instigating a multitude of defense mechanisms against plant early blight pathogens [25]. The amplification of this advantageous fungus through the administration of small peptides could potentially suppress pathogen infection and support healthy plant growth and development [26]. Conversely, although no studies have directly shown that Ramularia can cause tea plant diseases, studies have shown that Ramularia can cause diseases in many plants, including but not limited to barley, beets and strawberries [27, 28]. Therefore, this risk can be given due attention when using small peptides as foliar fertilizers. Small peptides significantly inhibited the proliferation of *Plectosphaerella* and *Coniothyrium* compared to the control group. *Plectosphaerella* is a plant pathogen that can cause organ rot and result in significant agricultural losses [29, 30], while *Coniothyrium* is also a plant pathogen that poses a potential threat to agricultural production [31, 32]. Small peptides may possess potential inhibitory effects on *Plectosphaerella* and *Coniothyrium* compared to the control group indicates their potential role in suppressing plant pathogenic fungi.

As a chemical surfactant, Tween 20 significantly inhibited the diversity of bacterial and fungal communities in the tea phyllosphere. The addition of Tween 20 to small peptides resulted in a reduction of 50 OTUs specific to bacterial communities and 251 OTUs specific to fungal communities. The impact of Tween 20 on fungal populations was more significant. The use of Tween 20 resulted in the dominance of *Paracoccus, unidentified Halomonadaceae*, and *Brevundimonas* as bacterial populations, and the dominance of *Cyphellophora, Debaryomyces, Cryptococcus, Pseudopestalotiopsis*, and *Phallus* as fungal populations. *Brevundimonas* has the potential to improve plant growth and promote nitrogen uptake [33]. *Debaryomyces* can be used for surfactant production, enhance oxidative stress tolerance, and serve as a biological control agent against post-harvest fruit diseases. It also has the potential to help plants resist anthracnose disease [34–36]. However, it should be noted that *Cyphellophora* is a plant pathogenic fungus [37], and specifically, *Pseudopestalotiopsis* is a pathogen that can cause grey blight disease of tea [38–40]. Therefore, based on this study, Tween 20 may not be suitable for use in tea plants, but further research is needed to prove this conclusion.

As a biological surfactant, rhamnolipid significantly inhibited the diversity of bacterial and fungal communities in the tea phyllosphere. The addition of rhamnolipid to small peptides resulted in a reduction of 113 OTUs specific to bacterial communities and 257 OTUs specific to fungal communities. The impact of rhamnolipid on fungal populations was more significant. The use of rhamnolipid resulted in the dominance of *Pseudomonas* and *Chryseobacterium* as bacterial populations, and the dominance of *Meyerozyma, Zasmidium, Vishniacozyma, Penicillium*, and *Aspergillus* as fungal populations. *Pseudomonas* has the potential to act as a plant biostimulant and improve drought resistance in rapeseed [41, 42]. Studies have shown that *Chryseobacterium* in the rice phyllosphere has a role in suppressing rice blast disease [43], and *Chryseobacterium* isolated from potato tissue can act as a biocontrol agent against bacterial wilt disease [44]. *Meyerozyma* is a beneficial plant-microbe that promotes plant growth [45], triggers plant immune responses [46] and has multiple beneficial effects such as preventing post-harvest decay and insect pest control in fruits [47, 48]. *Vishniacozyma* has a biocontrol effect against plant diseases caused by fungi [49, 50] and can inhibit plant decay [51]. All indications indicate that the combination of small peptides and rhamnolipid is the optimal combination for this study.

The addition of sophorolipid to small peptides resulted in an increase of 48 OTUs specific to bacterial communities and a reduction of 156 OTUs specific to fungal communities in the tea phyllosphere. The impact of sophorolipid on fungal populations was more significant. The use of sophorolipid resulted in the dominance of *Acinetobacter, Massilia, Truepera*, and *Cutibacterium* as bacterial populations, and the dominance of *Pezoloma, Diaporthe, Phialocephala*, and *Lichenomphalia* as fungal populations. *Acinetobacter* has the potential to promote plant growth by secreting plant growth hormone-like substances [52]. *Massilia* is a chloroacetamide herbicide-degrading bacterium [53]. *Pezoloma* may have the potential to alleviate plant drought stress [54], and *Phialocephala* can suppress some plant pathogens [55]. However, *Diaporthe* is frequently reported as a plant pathogen that can infect various plants and cause significant crop diseases. Therefore, when using sophorolipid, attention should be paid to the prevention and control of this pathogen [56–58].

## Conclusions

The use of small peptides and surfactants as common additives in foliar fertilizers significantly affected the structure of the tea phyllosphere microbial community. The use of small peptides reduced the diversity of bacterial communities in the tea phyllosphere and increased the diversity of fungal communities. The use of surfactants influenced the diversity of bacteria and fungi. However, the optimal combination in this experiment was found to be small peptides and rhamnolipid. The addition of rhamnolipid to small peptides resulted in the dominance of several beneficial microbes, such as *Pseudomonas, Chryseobacterium, Meyerozyma*, and *Vishniacozyma*, which may have the potential to promote the growth of tea plants and improve the immune response of tea plants. It is essential to consider the impact of foliar fertilizers on the tea phyllosphere microbial community and their function to ensure stability and sustainability during their application. The results of this study can provide a reference for the regulation of the phyllosphere microbial community in tea plant cultivation management. However, we acknowledge that these findings are based on a single experiment and thus recommend additional research to validate and expand upon our results.

## Materials and methods

### Experimental materials

Two-year-old national excellent variety “Longjing 43” tea plants were planted in nutrient pots with suitable cultivation substrates and were grown in a controlled environment chamber (planted on July 21, 2022). The organic matter content of the cultivation substrate was about 60%, the total porosity was about 75%, the bulk density was about 0.35%, and the pH was about 5.5. The tea plants were maintained under the following growth conditions: 14 h of daylight and 10 h of darkness, light intensity of 1000 lx, and air humidity of 70%. 

### Experimental treatments

After seven days of adaptation to an environment of 28℃ during the day and 22℃ at night, tea plants were subjected to the following treatments (Table 1): foliar spraying with pure water (CK), 6 g L^− 1^ of small peptides (P0), 6 g L^− 1^ of small peptides and 4‰ Tween-20 chemical surfactant (PT), 6 g L^− 1^ of small peptides and 4‰ rhamnolipid (PR), and 6 g L^− 1^ of small peptides and 4‰ sophorolipid (PS). Dose selection criteria refer to previous studies [10]. Small peptides were provided by Shandong Tianjiu Biotechnology Co., Ltd. (Heze, China). Tween-20 was provided by Tianjin Beilian Fine Chemicals Development Priority Co., Ltd. (Tianjin, China). Rhamnolipid was provided by Shaanxi Ruijie Biotechnology Co., Ltd. (Xi ‘an, China). Sophorolipid is provided by Shandong Yousuo Chemical Technology Co., Ltd (Heze, China). The treatments were applied every seven days during the experimental period. Each treatment had a total of 60 tea plants, and each treatment was divided into 3 groups, with 20 plants in each group. Tea phyllosphere samples were collected for bacterial and fungal community analysis four days after second treatment. The first mature leaf under the new shoots of the same tea tree was selected for analysis, and 20 leaves at the same leaf position were selected for each sample.

**Table 1:** The treatment methods in the test

Group	Treatments
CK	pure water
P0	small peptides
PT	small peptides and Tween-20
PR	small peptides and rhamnolipid
PS	small peptides and sophorolipid

### Sequencing analysis of Phyllosphere Surface Bacterial and Fungal Communities

In this experiment, 16S amplicon sequencing was used to sequence the bacterial community [59–61], and ITS amplicon sequencing was used to sequence the fungal community [62, 63]. Each treatment was repeated three times.

### Genomic DNA extraction and PCR amplification

Genomic DNA was extracted from the samples using the CTAB method, and the purity and concentration of the DNA were determined by agarose gel electrophoresis [64]. The extracted DNA was diluted to a concentration of 1ng µl^− 1^ with sterile water, and specific primers with barcodes, Phusion® High-Fidelity PCR Master Mix with GC Buffer from New England Biolabs, and a high-efficiency, high-fidelity enzyme were used for PCR amplification to ensure amplification efficiency and accuracy. The DNA samples underwent individual amplification within the V4 hyper variable regions via the utilization of PCR employing primers 515 F and 806R for 16 S rDNA in bacteria, and primers ITS5-1737 F and ITS2-2043R for ITS in fungi [65].

### PCR product mixing and purification

PCR products were detected by 2% agarose gel electrophoresis, and the qualified PCR products were purified using magnetic beads. After enzyme quantification, the PCR products were mixed in equal amounts according to the concentration of the PCR products, thoroughly mixed, and then subjected to 2% agarose gel electrophoresis again. The target bands of the PCR products were recovered using the gel recovery kit provided by Qiagen.

### Library Construction and sequencing

The TruSeq® DNA PCR-Free Sample Preparation Kit was used for library construction, and the constructed library was quantified using Qubit and Q-PCR. The library was sequenced using the NovaSeq6000.

### Sequencing data Processing and Analysis

The raw sequencing data were processed using the interactive Metware Microbial Diversity Analysis Cloud platform (https://cloud.metware.cn/, Metware Biotechnology Co., Ltd., Wuhan, China). After splicing, filtering, and de-chimerization, the data were subjected to Operational Taxonomic Units (OTUs) clustering, species annotation, α-diversity analysis, and β-diversity analysis. The bacterial function was predicted using Tax4Fun2 [66], and the fungal function was predicted using FunGuild [67].

### Electronic supplementary material

Below is the link to the electronic supplementary material.

Supplementary Material 1

## Data Availability

The raw sequencing data were deposited in NCBI Sequence Read Archive (SRA) under accession number PRJNA952937 for bacteria and PRJNA953843 for fungi.

## List of Abbreviations

CK: Foliar spraying with pure water
P0: Foliar spraying with small peptides
PT: Foliar spraying with small peptides and Tween-20
PR: Foliar spraying with small peptides and rhamnolipid
PS: Foliar spraying with small peptides and sophorolipid
OTUs: Operational taxonomic units
