# Supplementary material for: Improved phyllosphere microbiome composition of tea plant with the application of small peptides in combination with rhamnolipid
Source: BMC Microbiol. 2023 Oct 23;23:302. doi: 10.1186/s12866-023-03043-0 (PMC10591406; doi:10.1186/s12866-023-03043-0)

Figure S1 The Shannon index of bacterial community (A), Shannon index of fungal community (B); Chao1 index of bacterial community (C), Chao1 index of fungal community (D); UPGMA cluster tree of bacteria (E), UPGMA cluster tree of fungi (F). "ns" indicates not significant ($p \geqslant 0.05$), "*" indicates significant ($p < 0.05$), "**" indicates highly significant ($p < 0.01$), "***" indicates extremely significant ($p < 0.001$).

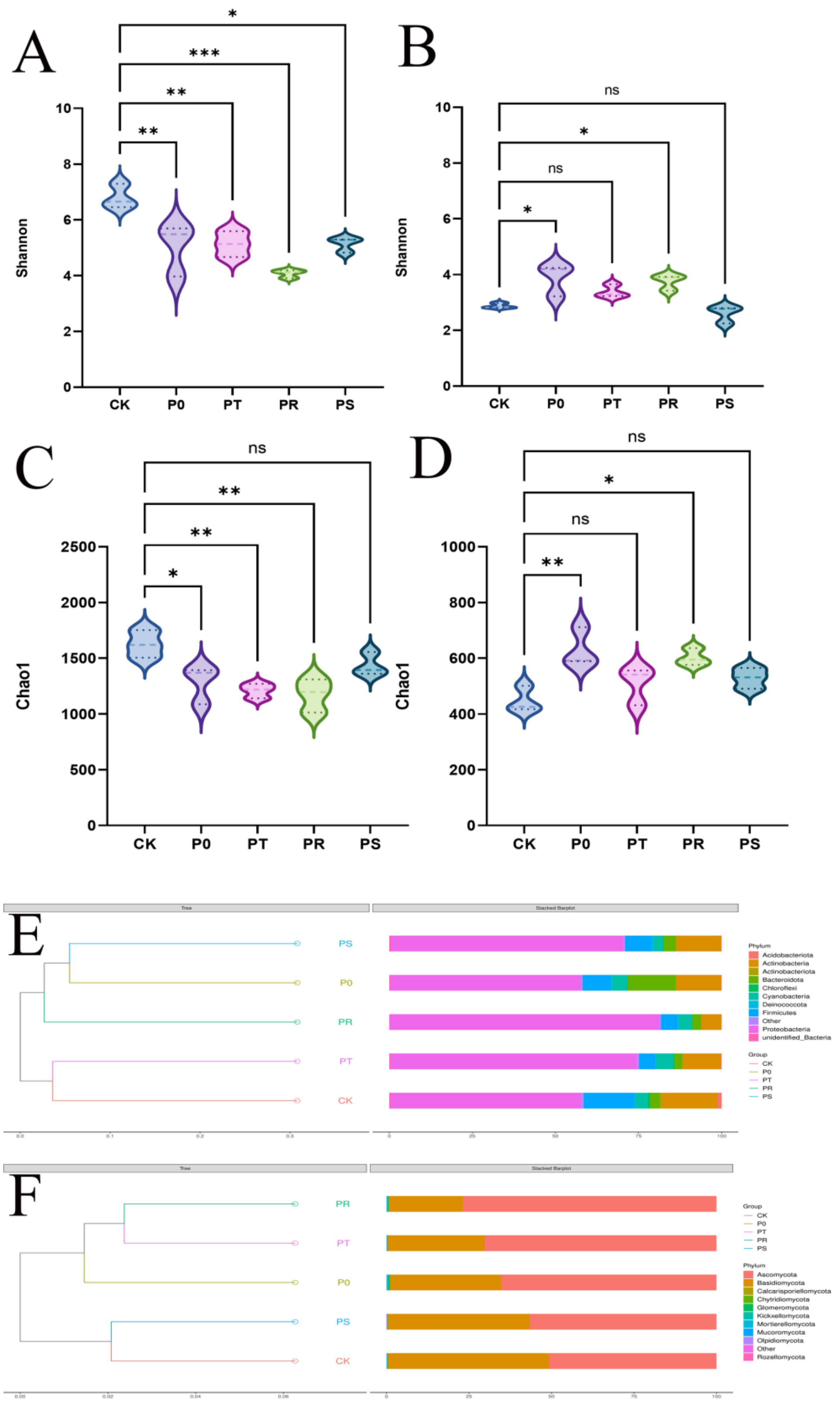

Supplement: Supplementary file 1 — Supplementary Material 1 [file 12866_2023_3043_MOESM1_ESM.pdf]
